# Influences of resolvin D1 and D2 on the risk of type 2 diabetes mellitus: a Chinese community-based cohort study

**DOI:** 10.3389/fimmu.2023.1143456

**Published:** 2023-06-02

**Authors:** Qian Sun, Junrong Wang, Yang Jing, Jingchao Liu, Jianrong Jin, Sudan Wang, Jin Zhang, Kaili Liu, Xiaofang Chen, Hui Zhou, Chen Dong

**Affiliations:** ^1^ Department of Epidemiology and Statistics, School of Public Health, Medical College of Soochow University, Soochow, Jiangsu, China; ^2^ Division of Non-communicable Diseases, Suzhou Industrial Park Centers for Disease Control and Prevention, Soochow, China; ^3^ Division of Non-communicable Diseases, Suzhou Wuzhong Centers for Disease Control and Prevention, Soochow, China

**Keywords:** type 2 diabetes mellitus, resolvin D1, resolvin D2, cohort study, association

## Abstract

**Background:**

Although cellular and animal studies have reported that resolvin D1 (RvD1) and resolvin D2 (RvD2) are mechanisms involved in the development of type 2 diabetes mellitus (T2DM), the impact of RvD1 and RvD2 on the risk of T2DM at a population level remains unclear.

**Methods:**

We included 2755 non-diabetic adults from a community-based cohort in China and followed them for seven years. Cox proportional hazards model was used to estimate hazard ratios (HRs) and 95% confidence intervals (CIs) for the association of RvD1 and RvD2 with T2DM probability. Time-dependent receiver operator characteristics (ROC) curve was used to evaluate the predictive performance of RvD1 and RvD2 for the risk of T2DM based on the Chinese CDC T2DM prediction model (CDRS).

**Results:**

A total of 172 incident T2DM cases were identified. Multivariate-adjusted HRs (95% CI) for T2DM across quartiles of RvD1 levels (Q1, Q2, Q3 and Q4) were 1.00, 1.64 (1.03-2.63), 1.80 (1.13-2.86) and 1.61 (1.01-2.57), respectively. Additionally, body mass index (BMI) showed a significant effect modification in the association of RvD1 with incident T2DM (*P*_interaction_ = 0.026). After multivariate adjustment, the HR (95% CI) for T2DM in the fourth compared with the first quartile of RvD2 was 1.94 (95% CI: 1.24-3.03). Time-dependent ROC analysis showed that the area under time-dependent ROC curves of the “CDRS+RvD1+RvD2” model for the 3-, 5- and 7-year risk of T2DM were 0.842, 0.835 and 0.828, respectively.

**Conclusions:**

Higher RvD1 and RvD2 levels are associated with a higher risk of T2DM at the population level.

## 1 Introduction

Despite the efforts to combat type 2 diabetes mellitus (T2DM), the rapid growth in its prevalence remains a massive challenge worldwide. According to the report by the International Diabetes Federation, the number of T2DM patients will increase to about 744 million worldwide by 2045 (1). It is now widely recognized that chronic low-grade inflammation plays a critical role in the development, progression and long-term complications of T2DM (2–4). Compared to the healthy controls, T2DM patients have significantly elevated levels of inflammation markers such as interleukin-6 (IL-6) and high-sensitivity C-reactive protein (hs-CRP) (5–7). In general, the inflammatory response of an organism is maintained by a balance between the occurrence and resolution of inflammation in physiological conditions (8, 9). Therefore, there is an increasing interest in the potential role of inflammation resolution in T2DM development.

The resolution of inflammation is a critical endogenous process that protects host tissues from prolonged or excessive inflammation that can become chronic (10). Resolvin D1 (RvD1) and resolvin D2 (RvD2), two important members of the family of specialized pro-resolving mediators (SPMs), are generated from the n-3 polyunsaturated fatty acids docosahexaenoic acid (DHA) (11, 12). Over the past decade, more and more studies have suggested that both RvD1 and RvD2 exert potent counter-regulatory effects on pro-inflammatory signaling pathways, acting as “brakes” on the persistent vicious cycle leading to unremitting inflammation (13, 14). For example, Claria et al. reported that RvD1 and RvD2 are potent pro-resolving mediators in counteracting local adipokine production and monocyte accumulation in obesity-induced adipose inflammation (15). RvD1 and RvD2 have the ability not only to rescue impaired expression and secretion of adiponectin in inflamed obese adipose tissue, but also to reduce the production of pro-inflammatory adipokine such as leptin. Moreover, the results from cellular studies and animal model have found that the production of RvD1 and RvD2 is significantly reduced under diabetes or high glucose culture conditions (16, 17).

Although cellular and animal studies showed that RvD1 and RvD2 have a beneficial effects on the T2DM development (18, 19), the prospective impact of RvD1 and RvD2 on the risk of T2DM at the population level has not been reported to date. In an effort to address this issue, this study aims to examine the association of plasma RvD1 and RvD2 concentrations with future T2DM probability based on a community-based Chinese cohort.

## 2 Materials and methods

### 2.1 Study population

“The prevention of MS and multi-metabolic disorders in Jiangsu province of China II (PMMJS-II)” is a community-based prospective cohort study in Soochow, China. The details of the study have been previously described (20, 21). Briefly, 3700 participants aged 35-60 years were recruited from Soochow (China) by a multistage sampling method between 06/2014 and 05/2015. Follow-up examination was performed every two years. To characterize the plasma RvD1 and RvD2 levels at baseline and to test their effects on the subsequent 7-year risk of T2DM, individuals who had the following conditions were excluded: 1) type 1 and 2 diabetes mellitus diagnosed before 2015 (639); 2) chronic hepatitis B and hepatitis C, schistosomiasis, tuberculosis, acquired immune deficiency syndrome and other severe chronic communicable diseases (189); 3) severe psychological disorders, physical disabilities, and cancer within 6 months (18). After further excluding 99 individuals whose plasma sample is not enough, 2755 participants were ultimately included in the current analysis. This study was approved by the ethics committee of Suzhou Industrial Park Centers for Disease Control and Prevention in accordance with the Declaration of Helsinki. All participants gave written informed consent.

### 2.2 Plasma RvD1 and RvD2 measurement

Blood sample was drawn from each participant after fasting at least 8 hours and the serum and plasma samples were separated immediately. RvD1 and RvD2 concentration were assessed in plasma sample using the human RvD1 and RvD2 enzyme linked immunosorbent assay (ELISA) kits according to the standard protocol (Cayman Chemical Co., Ann Arbor, MI, United States), respectively. The optical density was recorded with a plate reader (Bio-Tek) and the RvD1 and RvD2 levels were calculated using the standard curve. Based on manufacturing criteria, the detection sensitivity of the RvD1 assay was 15 pg/ml, as did RvD2 assay.

Participants were divided by quartiles (Q1-4) of the baseline level of RvD1 and RvD2, with < 32.16 pg/mL, 32.17-51.76 pg/mL, 51.77-91.92 pg/mL and ≥ 91.93 pg/mL for RvD1, and < 45.83 pg/mL, 45.84-82.67 pg/mL, 82.68-130.10 pg/mL and ≥ 130.11 pg/mL for RvD2, respectively. The optimal cutoff values of RvD1 and RvD2 were respectively 33.27 and 110.75 pg/mL for predicting T2DM, determined by the receiver operating characteristic (ROC) curve.

### 2.3 Assessment of T2DM

Diagnosis of T2DM was made based on established criteria (ICD-10 code E11; http://apps.who.int/classifications/icd10/browse/2016/en) as following: (a) FPG ≥ 7.0 mmol/L or HbA1c ≥ 6.5%; (b) 2-hour oral glucose tolerance test ≥ 11.1 mmol/L; (c) Use of antidiabetic drugs; (d) Self-report of T2DM diagnosed by physician. All cases that were diagnosed as type 1 diabetes, secondary diabetes, or others types of diabetes were excluded. The cohort was followed from the baseline date in 2014-2015 to the date of incidence of T2DM, death, or until the end of the observation (31 December 2021), whichever came first.

### 2.4 Covariates

Based on the previous studies, several risk factors that could potentially confound the association of RvD1 and RvD2 with T2DM risk were collected by trained interviewers and assumed to be covariates, including demographic variables (age, gender, body weight, height and etc.), life style factors (smoking and alcohol consumption), disease history (hypertension, dyslipidemia and etc.), and medical history (antihypertensive medication and lipid-lowering drug). Height, weight, and blood pressure were measure and recorded according to standard procedures. Fasting serum total cholesterol (TC), triglycerides (TG), high-density lipoprotein cholesterol (HDL-c) and fasting plasma glucose (FPG) were measured using an automated analyzer. The low-density lipoprotein cholesterol (LDL-c) level was calculated using the Friedewald equation. Plasma level of hs-CRP was determined using a chemiluminescent immunoassay. Hypertension was defined as systolic blood pressure (SBP) ≥140 mmHg, diastolic blood pressure (DBP) ≥90 mmHg, or use of antihypertensive medications. Dyslipidemia was defined as TG ≥ 1.7 mmol/L and/or TC ≥ 5.18 mmol/L and/or LDL-c ≥ 3.37 mmol/L and/or HDL-c ≤1.04 mmol/L, or use of lipid-lowering drug.

### 2.5 Statistical analysis

Data were presented as mean ± standard deviation (SD) or median (interquartile range) for continuous variables and as number (percentage) for categorical variables. The unpaired *t*-test, *χ^2^* test or Fisher’s exact test was used to compare differences of continuous variables and categorical variables, as appropriate. Multivariable linear regression analyses were used to test the correlation of RvD1 or RvD2 with the baseline variables including age, body mass index (BMI), SBP, DBP, FPG, TC, TG, LDL-c, HDL-c and hs-CRP, and the correlation between RvD1 and RvD2 concentrations.

Cumulative incidences of T2DM were visualized using Kaplan-Meier plots stratified by quartiles of RvD1 and RvD2 concentrations, respectively. Differences between cumulative incidences for RvD1 and RvD2 quartiles were tested for statistical significance using the log-rank test. The linearity of RvD1 or RvD2 for the risk of T2DM was examined using the restricted cubic splines Cox model. Analyses were multivariable-adjusted using 5 knots (located at the 5th, 25th, 50th, 75th, and 95th percentiles) that could provide an adequate fit of the model (22). In this study, Cox proportional hazards models were used to estimate hazard ratios (HRs) and 95% confidence intervals (CIs) for the association of RvD1 and RvD2 with future T2DM probability, regarding the first quartile of RvD1 or RvD2 as reference. In addition, we also analyzed the association of RvD1 and RvD2 with incident T2DM by comparing RvD1 ≥33.27 pg/mL to < 33.27 pg/mL, and RvD2 ≥110.57 pg/mL to < 110.57 pg/mL, respectively. Covariates of the models were selected as described above. In addition to the unadjusted model, results for the minor adjustment model (model 1, adjustment for age, gender, BMI, smoking, alcohol consumption and family history of diabetes) and full adjustment model (model 2, model 1 plus adjustment for dyslipidemia, and hs-CRP) were presented. A series of sensitivity analyses based on model 2 were performed to test the robustness of the results: 1) exclude the population whose BMI ≥ 28 kg/m^2^; 2) exclude people who using lipid-lowering drug; 3) To avoid the reverse effect of T2DM on the production of RvD1 and RvD2, the participants who developed T2DM in the first year of follow-up (2016) were excluded; 4) To avoid the interaction between RvD1 and RvD2, we additionally adjusted RvD1 for the association of RvD2 with T2DM, and additionally adjusted RvD2 for the association of RvD1 with T2DM, respectively.

To detect effect modification, we performed subgroup analyses according to baseline characteristics (age, gender, BMI, smoking status, alcohol consumption, dyslipidaemia and family history of diabetes). Possible interactions between RvD1 or RvD2 and risk factors, with respect to T2DM incidence, were tested by introducing interaction terms into the multivariate model (one at a time). To quantify the RvD1 or RvD2 for the future T2DM prediction, we drew time-dependent ROC curves and calculated the area under the time-dependent ROC curves (AUROCs) for the 3-year, 5-year, and 7-year risk of T2DM based on the traditional risk factors described in the Chinese Centre for Disease Control and Prevention (CDC) T2DM prediction model (CDRS), and calculated the corresponding best threshold. SAS version 9.4 (SAS Institute, Cary, NC, USA) and R 4.0.2 (R Foundation for Statistical Computing, Vienna, Austria) were used for the data analysis. All *P*-values are two-tailed and statistical significance was set at *P*<0.05.

## 3 Results

**Table 1** showed the baseline characteristics of the study population stratified by quartiles of plasma RvD1 and RvD2 concentrations, respectively. The prevalence of males, smokers and alcohol consumers were increased with RvD1 concentration. In addition, individuals with higher RvD1 levels were more likely to have higher levels of SBP and DBP, as well as elevated TG, TC and LDL-c concentrations. As the results shown, participants with higher RvD2 levels were more likely to be older, to drink alcohol, and to have higher levels of FPG, TG, TC, LDL-c and lower HDL-c concentrations.

**Table 1:** Baseline characteristics of the study population stratified according to the quartiles of RvD1 and RvD2.

	RvD1 subgroup, pg/ml				*P*	RvD2 subgroup, pg/ml				*P*
Variables	Q1 (<32.16)	Q2 (32.16-51.76)	Q3 (51.77-91.92)	Q4 (≥91.93)		Q1 (<45.83)	Q2 (45.83-82.67)	Q3 (82.68-130.10)	Q4 (≥130.11)	
N (case)	691 (28)	687 (47)	689 (49)	688 (48)		688 (30)	689 (40)	689 (46)	689 (56)	
Age (years)	49.92 ± 6.33	50.01 ± 6.11	49.83 ± 5.88	49.90 ± 5.67	0.941	49.32 ± 5.61	49.90 ± 6.34	50.06 ± 6.24	50.38 ± 5.73	0.020
Male, n (%)	229 (33.14)	272 (39.59)	256 (37.16)	302 (43.90)	0.001	285 (41.42)	257 (37.30)	258 (37.45)	259 (37.59)	0.325
BMI (kg/m²)	23.68 ± 2.92	23.95 ± 3.13	23.82 ± 3.03	23.91 ± 2.74	0.148	23.75 ± 3.02	23.79 ± 3.03	23.96 ± 2.90	23.85 ± 2.88	0.478
Smoking, n (%)	152 (22.00)	177 (25.76)	169 (24.53)	200 (29.07)	0.024	192 (27.91)	168 (24.38)	165 (23.95)	173 (25.11)	0.327
Alcohol consumption, n (%)	104 (15.05)	119 (17.32)	124 (18.00)	157 (22.82)	0.002	141 (20.49)	106 (15.38)	116 (16.84)	141 (20.46)	0.026
Family history of diabetes, n (%)	89 (12.88)	90 (13.10)	74 (10.74)	88 (12.79)	0.512	96 (13.95)	75 (10.89)	83 (12.05)	87 (12.63)	0.377
SBP (mmHg)	122.37 ± 14.56	122.73 ± 16.14	124.77 ± 15.09	125.68 ± 14.92	<0.001	123.70 ± 15.23	123.03 ± 14.83	123.85 ± 14.64	124.98 ± 16.20	0.175
DBP (mmHg)	76.39 ± 11.19	76.74 ± 11.93	78.11 ± 11.14	78.62 ± 10.90	<0.001	77.40 ± 11.39	76.85 ± 11.13	77.47 ± 10.90	78.17 ± 11.86	0.259
HTN, n (%)	237 (34.30)	249 (36.24)	277 (40.20)	299 (43.46)	0.002	251 (36.48)	250 (36.28)	263 (38.17)	298 (43.25)	0.027
Lipid-lowering drug, n (%)	4 (0.58)	3 (0.44)	6 (0.87)	6 (0.87)	0.699	6 (0.87)	3 (0.44)	6 (0.87)	4 (0.58)	0.698
FPG (mmol/L)	5.45 ± 0.36	5.45 ± 0.42	5.48 ± 0.44	5.49 ± 0.38	0.362	5.45 ± 0.33	5.46 ± 0.41	5.48 ± 0.41	5.50 ± 0.44	0.024
TG (mmol/L)	1.22 ± 0.65	1.40 ± 0.86	1.52 ± 1.09	1.70 ± 1.30	<0.001	1.32 ± 0.88	1.34 ± 0.85	1.46 ± 0.93	1.70 ± 1.28	<0.001
TC (mmol/L)	4.78 ± 0.89	4.86 ± 0.94	4.80 ± 0.85	4.97 ± 0.91	0.001	4.76 ± 0.84	4.84 ± 0.90	4.85 ± 0.90	4.96 ± 0.95	0.005
LDL-c (mmol/L)	2.97 ± 0.71	3.07 ± 0.78	2.97 ± 0.70	3.09 ± 0.78	0.004	2.88 ± 0.67	3.04 ± 0.71	3.02 ± 0.74	3.15 ± 0.82	<0.001
HDL-c (mmol/L)	1.25 ± 0.33	1.20 ± 0.29	1.22 ± 0.26	1.24 ± 0.29	0.053	1.25 ± 0.28	1.22 ± 0.31	1.24 ± 0.29	1.20 ± 0.28	0.006
Dyslipidemia, n (%)	420 (60.78)	441 (64.19)	438 (63.57)	487 (70.78)	0.001	427 (62.06)	442 (64.15)	443 (64.30)	474 (68.80)	0.063
hs-CRP (mg/L)	1.80 ± 2.10	1.66 ± 1.57	1.76 ± 2.18	1.82 ± 2.39	0.086	1.74 ± 2.41	1.81 ± 2.28	1.72 ± 1.66	1.76 ± 1.90	0.049

RvD1, resolvin D1; RvD2, resolvin D2; Q1, quartile 1; Q2, quartile 2; Q3, quartile 3; Q4, quartile 4; BMI, body mass index; SBP, systolic blood pressure; DBP, diastolic blood pressure; HTN, hypertension; FPG, fasting plasma glucose; TG, triglycerides; TC, total cholesterol; LDL-c, low-density lipoprotein cholesterol; HDL-c, high-density lipoprotein cholesterol; hs-CRP, high-sensitivity C-reactive protein.

**Figure 1:**
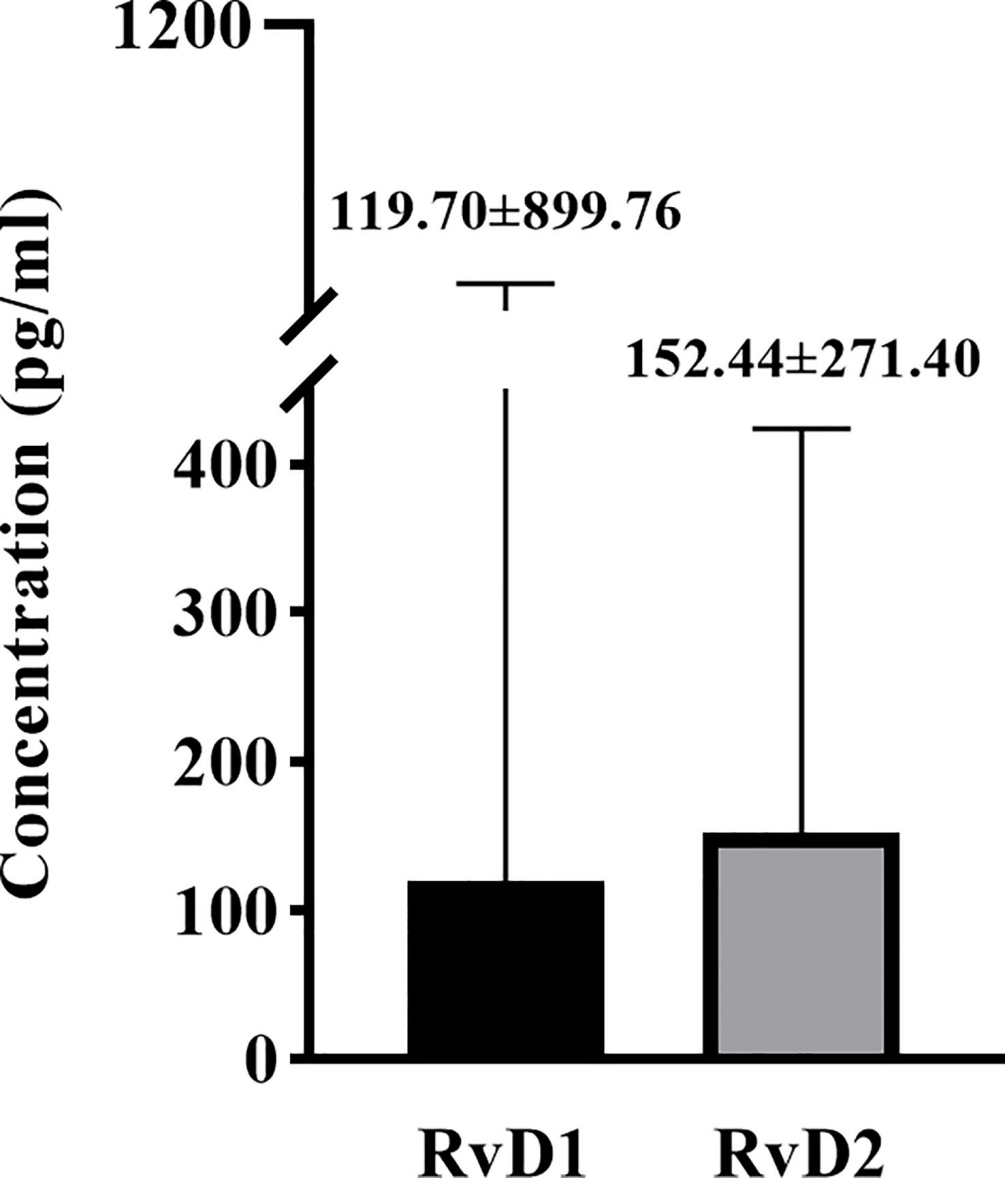
The baseline levels of plasma RvD1 and RvD2 in the study population.

**Figure 2:**
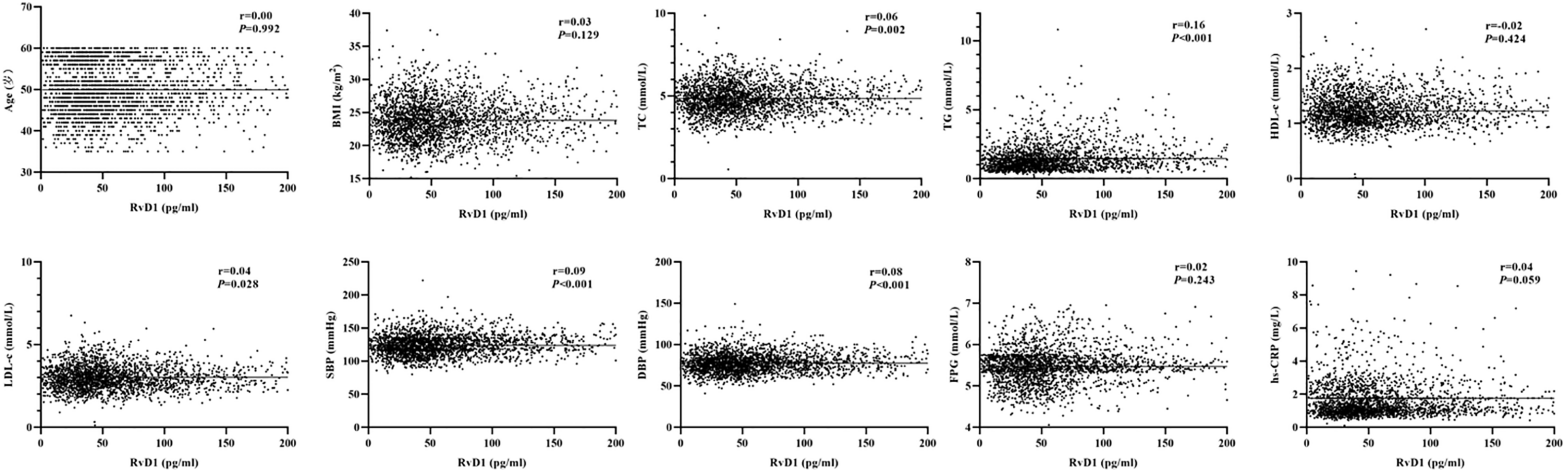
Analysis on the correlations between plasma RvD1 levels and the characteristics of study population.

**Figure 3:**
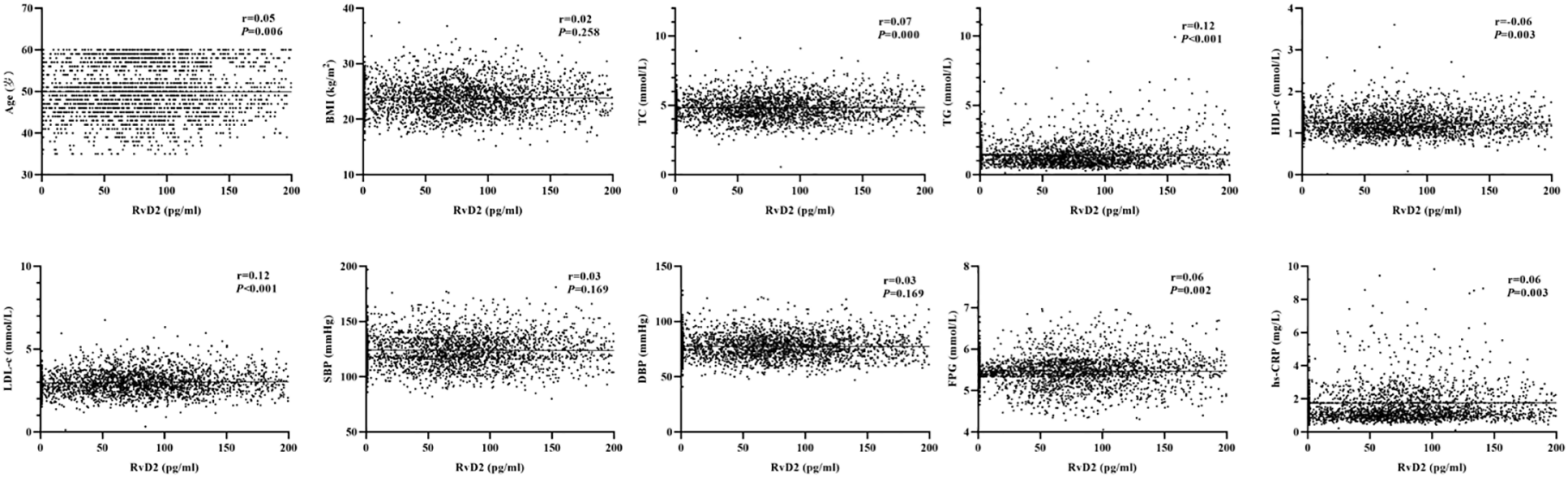
Analysis on the correlations between plasma RvD2 levels and the characteristics of study population.

As shown in **Figure 1**, the baseline levels of plasma RvD1 and RvD2 in the study population were 119.70 ± 899.76 pg/mL and 152.44 ± 271.40 pg/mL, respectively. The plasma RvD1 concentration was significantly correlated with SBP and DBP levels, as well as serum TC, TG and LDL-c concentrations (all *P* < 0.05) (**Figure 2**). The plasma concentration of RvD2 was positively correlated with age, and serum levels of TC, TG, LDL-c and FPG concentration, but negatively correlated with HDL-c concentration (all *P* < 0.05) (**Figure 3**). In addition, the plasma RvD1 concentration was significantly correlated with the RvD2 concentration (*r* = 0.28, *P* < 0.001). Of note, baseline hs-CRP levels were positively correlated with plasma RvD2 levels (*r* = 0.06, *P* = 0.003).

During the seven-year follow-up period, 172 new cases of T2DM were documented (incidence density: 10.09 per 1,000 person-years). Cumulative incidences of T2DM stratified by quartiles of RvD1 and RvD2 concentrations were visualized using Kaplan–Meier plots (**Supplemental Figure 1**). Compared to those with lower plasma RvD1 levels, participants with higher RvD1 levels exhibited a higher risk of T2DM development (*P* = 0.046). Similarly, those with higher levels of RvD2 had an increased risk of developing T2DM (*P* = 0.015).

After adjustment for the potential confounders, the multivariate-adjusted HRs (95% CI) for T2DM across quartiles of RvD1 levels (Q1, Q2, Q3 and Q4) were 1.00, 1.64 (1.03-2.63), 1.80 (1.13-2.86) and 1.61 (1.01-2.57), respectively. As the results shown in **Supplemental Figure 2A**, there was a linear association between RvD1 concentrations and T2DM incidence (*P*_linearity_= 0.028). In model 1-2, RvD1 ≥ 33.27 pg/mL significantly increased the risk of T2DM compared with RvD1 < 33.27 pg/mL (HR: 1.79, 95% CI: 1.19-2.69) (**Table 2**). Results from sensitivity analyses showed that the association remained significant in the participants with RvD1 ≥ 33.27 pg/mL, even after excluding obese individuals, participants using lipid-lowering medications, new-onset T2DM in 2016, and additional adjustment for the baseline RvD2 (**Table 2**).

**Table 2:** Associations between the quartiles of RvD1 and the risk of T2DM.

	N (case)	Unadjusted HR (95% CI)	*P*	Adjusted HR (95% CI) in model 1	*P*	Adjusted HR (95% CI) in model 2	*P*
Quartiles of RvD1							
Q1 (<32.16 pg/ml)	691 (28)	1.00 (ref)		1.00 (ref)		1.00 (ref)	
Q2 (32.16-51.76 pg/ml)	687 (47)	1.76 (1.10-2.82)	0.018	1.63 (1.02-2.61)	0.040	1.64 (1.03-2.63)	0.038
Q3 (51.77-91.92 pg/ml)	689 (49)	1.81 (1.14-2.88)	0.012	1.82 (1.14-2.89)	0.012	1.80 (1.13-2.86)	0.014
Q4 (≥91.93 pg/ml)	688 (48)	1.72 (1.08-2.75)	0.022	1.66 (1.04-2.65)	0.034	1.61 (1.01-2.57)	0.047
* P_trend_*			0.054		0.075		0.084
RvD1 ≥33.27 pg/ml	2035 (144)	1.87 (1.25-2.80)	0.003	1.82 (1.21-2.74)	0.004	1.79 (1.19-2.69)	0.005
Sensitivity analysis							
Exclusion of people taking lipid-lowering drugs							
Q1 (<32.10 pg/ml)	684 (27)	1.00 (ref)		1.00 (ref)		1.00 (ref)	
Q2 (32.10-51.73 pg/ml)	684 (46)	1.78 (1.11-2.87)	0.017	1.64 (1.02-2.64)	0.042	1.66 (1.03-2.67)	0.038
Q3 (51.74-91.64 pg/ml)	684 (49)	1.88 (1.17-3.00)	0.009	1.86 (1.16-2.98)	0.010	1.84 (1.15-2.94)	0.011
Q4 (≥91.65 pg/ml)	684 (45)	1.67 (1.03-2.68)	0.036	1.59 (0.98-2.57)	0.058	1.54 (0.95-2.49)	0.077
* P_trend_*			0.051		0.074		0.080
RvD1 ≥33.27 pg/ml	2020 (140)	1.89 (1.25-2.85)	0.003	1.83 (1.21-2.77)	0.004	1.81 (1.19-2.73)	0.005
Exclusion of obese people							
Q1 (<31.96 pg/ml)	631 (21)	1.00 (ref)		1.00 (ref)		1.00 (ref)	
Q2 (31.96-52.07 pg/ml)	633 (39)	1.93 (1.13-3.27)	0.016	1.84 (1.08-3.14)	0.025	1.85 (1.09-3.16)	0.023
Q3 (52.08-92.94 pg/ml)	624 (39)	1.92 (1.13-3.27)	0.016	1.97 (1.16-3.36)	0.012	1.96 (1.15-3.34)	0.013
Q4 (≥92.95 pg/ml)	629 (44)	2.11 (1.26-3.55)	0.005	2.05 (1.21-3.46)	0.007	1.98 (1.17-3.35)	0.011
* P_trend_*			0.033		0.042		0.051
RvD1 ≥59.93 pg/ml	1077 (79)	1.64 (1.18-2.28)	0.003	1.64 (1.18-2.28)	0.004	1.60 (1.15-2.23)	0.006
Exclusion of new cases of diabetes in 2016							
Q1 (<32.10 pg/ml)	680 (20)	1.00 (ref)		1.00 (ref)		1.00 (ref)	
Q2 (32.10-51.79 pg/ml)	680 (37)	1.95 (1.13-3.36)	0.016	1.79 (1.04-3.08)	0.037	1.80 (1.04-3.10)	0.035
Q3 (51.80-91.52 pg/ml)	680 (43)	2.24 (1.32-3.81)	0.003	2.22 (1.30-3.77)	0.003	2.20 (1.29-3.74)	0.004
Q4 (≥91.53 pg/ml)	680 (37)	1.85 (1.07-3.18)	0.027	1.73 (1.00-2.99)	0.050	1.67 (0.97-2.89)	0.065
* P_trend_*			0.027		0.035		0.037
RvD1 ≥61.23 pg/ml	1130 (74)	1.63 (1.16-2.28)	0.005	1.61 (1.15-2.25)	0.006	1.57 (1.12-2.20)	0.009
Additional adjustments for baseline RvD2*							
Q1 (<32.16 pg/ml)	691 (28)	1.00 (ref)		1.00 (ref)		1.00 (ref)	
Q2 (32.16-51.76 pg/ml)	687 (47)	1.76 (1.10-2.81)	0.018	1.63 (1.02-2.61)	0.042	1.64 (1.03-2.62)	0.039
Q3 (51.77-91.92 pg/ml)	689 (49)	1.80 (1.13-2.87)	0.013	1.81 (1.13-2.87)	0.013	1.79 (1.12-2.85)	0.015
Q4 (≥91.93 pg/ml)	688 (48)	1.65 (1.01-2.70)	0.045	1.57 (0.95-2.57)	0.076	1.53 (0.93-2.51)	0.094
* P_trend_*			0.062		0.088		0.093
RvD1 ≥33.27 pg/ml	2035 (144)	1.85 (1.23-2.78)	0.003	1.79 (1.19-2.70)	0.005	1.77 (1.18-2.67)	0.006

Model 1: adjusted for age, gender, BMI, smoking, alcohol consumption, family history of diabetes.

Model 2: adjusted for age, gender, BMI, smoking, alcohol consumption, family history of diabetes, dyslipidemia, hs-CRP.

*Unadjusted model: adjusted for RvD2.

*Model 1: adjusted for age, gender, BMI, smoking, alcohol consumption, family history of diabetes, RvD2.

*Model 2: adjusted for age, gender, BMI, smoking, alcohol consumption, family history of diabetes, dyslipidemia, hs-CRP, RvD2.

RvD1, resolvin D1; RvD2, resolvin D2; T2DM, type 2 diabetes mellitus; Q1, quartile 1; Q2, quartile 2; Q3, quartile 3; Q4, quartile 4; HR, hazard ratio; CI, confidence interval.

Compared with participants in the lowest RvD2 quartile (quartile 1, Q1), those in the highest RvD2 quartile (quartile 4, Q4) had a higher risk of developing T2DM (HR: 1.94, 95% CI: 1.24-3.03) after adjustment for the potential confounders. Similarly, the participant with RvD2 ≥ 110.57 pg/mL had a higher risk of T2DM compared to those with RvD2 < 110.57 pg/mL (HR: 1.69, 95% CI: 1.25-2.28) (**Table 3**). The restricted cubic splines suggested a linear association between RvD2 concentrations and incident T2DM (*P*_linearity_= 0.003) (**Supplemental Figure 2B**). After excluding the participants with obesity, use of lipid-lowering medications, new-onset T2DM in 2016, or additional adjustment for the baseline RvD1, sensitivity analyses showed that plasma RvD2 remained significantly associated with the probability of future T2DM (**Table 3**).

**Table 3:** Associations between the quartiles of RvD2 and the risk of T2DM.

	N (case)	Unadjusted HR (95% CI)	*P*	Adjusted HR (95% CI) in model 1	*P*	Adjusted HR (95% CI) in model 2	*P*
Quartiles of RvD2							
Q1 (<45.83 pg/ml)	688 (30)	1.00 (ref)		1.00 (ref)		1.00 (ref)	
Q2 (45.83-82.67 pg/ml)	689 (40)	1.40 (0.87-2.25)	0.163	1.43 (0.89-2.29)	0.145	1.42 (0.88-2.28)	0.153
Q3 (82.68-130.10 pg/ml)	689 (46)	1.60 (1.01-2.54)	0.045	1.59 (1.00-2.52)	0.050	1.58 (0.99-2.50)	0.054
Q4 (≥130.11 pg/ ml)	689 (56)	2.01 (1.29-3.14)	0.002	1.98 (1.27-3.09)	0.003	1.94 (1.24-3.03)	0.004
* P_trend_*			0.019		0.026		0.033
RvD2 ≥110.57 pg/ml	924 (79)	1.76 (1.30-2.38)	<0.001	1.71 (1.27-2.32)	0.001	1.69 (1.25-2.28)	0.001
Sensitivity analysis							
Exclusion of people taking lipid-lowering drugs							
Q1 (<45.95 pg/ml)	684 (29)	1.00 (ref)		1.00 (ref)		1.00 (ref)	
Q2 (45.95-82.59 pg/ml)	684 (40)	1.46 (0.90-2.35)	0.124	1.48 (0.92-2.40)	0.108	1.48 (0.91-2.39)	0.113
Q3 (82.60-130.18 pg/ml)	684 (45)	1.63 (1.02-2.59)	0.042	1.62 (1.01-2.59)	0.044	1.62 (1.01-2.58)	0.045
Q4 (≥130.19 pg/ ml)	684 (53)	1.98 (1.26-3.11)	0.003	1.95 (1.24-3.07)	0.004	1.91 (1.21-3.02)	0.005
* P_trend_*			0.030		0.038		0.047
RvD2 ≥110.57 pg/ml	919 (76)	1.73 (1.27-2.34)	0.001	1.69 (1.24-2.29)	0.001	1.66 (1.22-2.25)	0.001
Exclusion of obese people							
Q1 (<45.35 pg/ml)	629 (24)	1.00 (ref)		1.00 (ref)		1.00 (ref)	
Q2 (45.35-82.50 pg/ml)	629 (33)	1.44 (0.85-2.43)	0.179	1.45 (0.86-2.46)	0.167	1.45 (0.85-2.46)	0.169
Q3 (82.51-130.26 pg/ml)	628 (37)	1.61 (0.96-2.69)	0.070	1.60 (0.95-2.67)	0.075	1.61 (0.96-2.70)	0.072
Q4 (≥130.27 pg/ml)	631 (49)	2.18 (1.34-3.56)	0.002	2.06 (1.26-3.37)	0.004	2.05 (1.25-3.36)	0.004
* P_trend_*			0.016		0.034		0.038
RvD2 ≥102.82 pg/ml	942 (72)	1.76 (1.27-2.45)	0.001	1.72 (1.24-2.39)	0.001	1.71 (1.23-2.38)	0.001
Exclusion of new cases of diabetes in 2016							
Q1 (<45.60 pg/ml)	680 (22)	1.00 (ref)		1.00 (ref)		1.00 (ref)	
Q2 (45.60-82.68 pg/ml)	680 (30)	1.45 (0.84-2.51)	0.188	1.51 (0.87-2.62)	0.145	1.51 (0.87-2.62)	0.147
Q3 (82.69-130.09 pg/ml)	681 (40)	1.92 (1.14-3.24)	0.014	1.95 (1.16-3.28)	0.012	1.94 (1.15-3.27)	0.013
Q4 (≥130.10 pg/ml)	679 (45)	2.25 (1.35-3.75)	0.002	2.25 (1.35-3.76)	0.002	2.21 (1.33-3.70)	0.002
* P_trend_*			0.012		0.013		0.016
RvD2 ≥94.10 pg/ml	1151 (77)	1.84 (1.31-2.58)	<0.001	1.82 (1.30-2.55)	0.001	1.79 (1.28-2.51)	0.001
Additional adjustments for baseline RvD1*							
Q1 (<45.83 pg/ml)	688 (30)	1.00 (ref)		1.00 (ref)		1.00 (ref)	
Q2 (45.83-82.67 pg/ml)	689 (40)	1.40 (0.87-2.25)	0.163	1.43 (0.89-2.29)	0.144	1.42 (0.88-2.28)	0.153
Q3 (82.68-130.10 pg/ml)	689 (46)	1.60 (1.01-2.54)	0.045	1.59 (1.00-2.52)	0.050	1.58 (0.99-2.50)	0.054
Q4 (≥130.11 pg/ml)	689 (56)	2.02 (1.29-3.15)	0.002	1.98 (1.26-3.09)	0.003	1.94 (1.24-3.03)	0.004
* P_trend_*			0.019		0.027		0.034
RvD2 ≥110.57 pg/ml	924 (79)	1.76 (1.30-2.39)	<0.001	1.71 (1.27-2.32)	0.001	1.69 (1.25-2.29)	0.001

Model 1: adjusted for age, gender, BMI, smoking, alcohol consumption, family history of diabetes.

Model 2: adjusted for age, gender, BMI, smoking, alcohol consumption, family history of diabetes, dyslipidemia, hs-CRP.

*Unadjusted model: adjusted for RvD1.

*Model 1: adjusted for age, gender, BMI, smoking, alcohol consumption, family history of diabetes, RvD1.

*Model 2: adjusted for age, gender, BMI, smoking, alcohol consumption, family history of diabetes, dyslipidemia, hs-CRP, RvD1.

RvD1, resolvin D1; RvD2, resolvin D2; T2DM, type 2 diabetes mellitus; Q1, quartile 1; Q2, quartile 2; Q3, quartile 3; Q4, quartile 4; HR, hazard ratio; CI, confidence interval.

**Figure 4:**
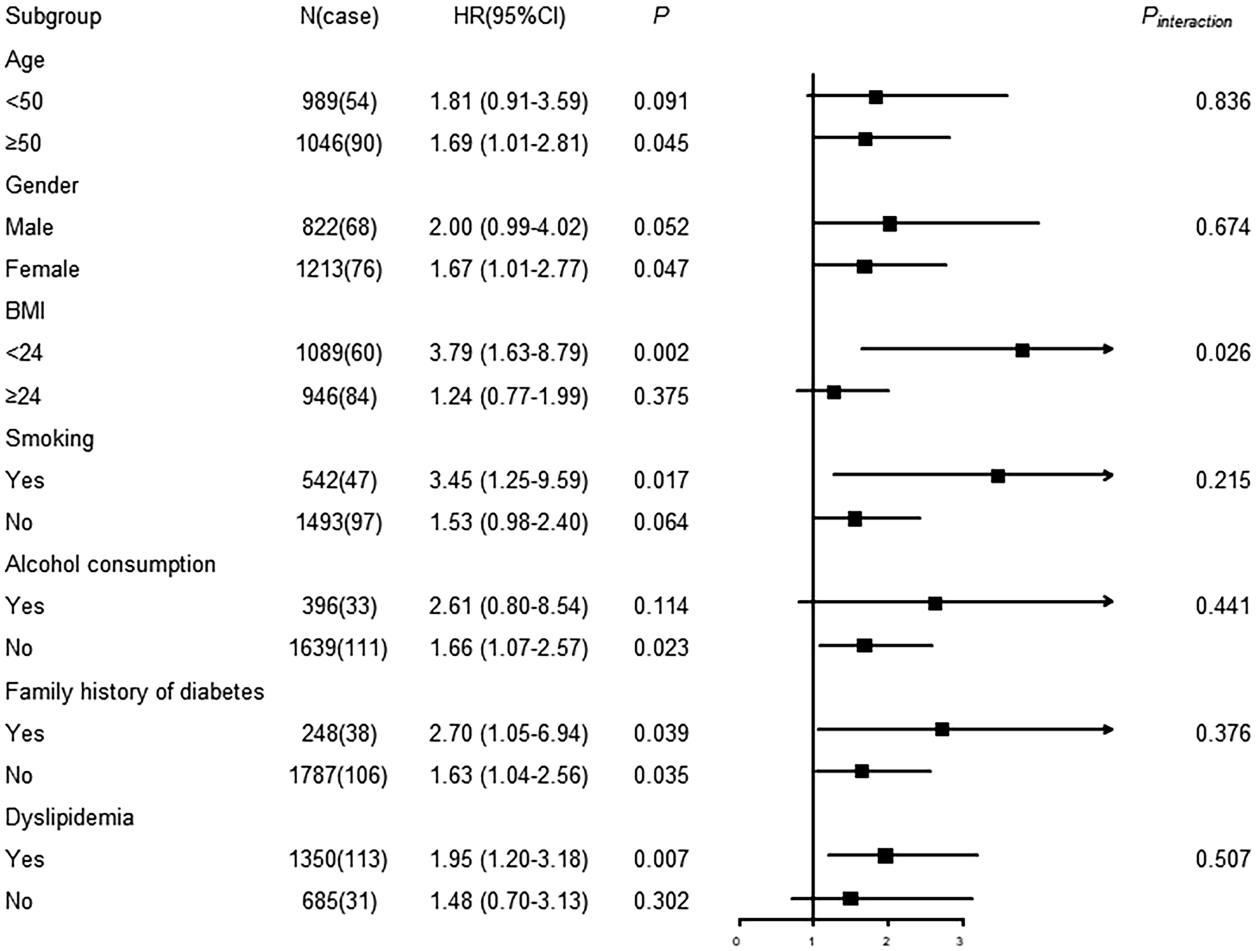
Subgroup analysis of associations between RvD1 and incident T2DM.

**Figure 5:**
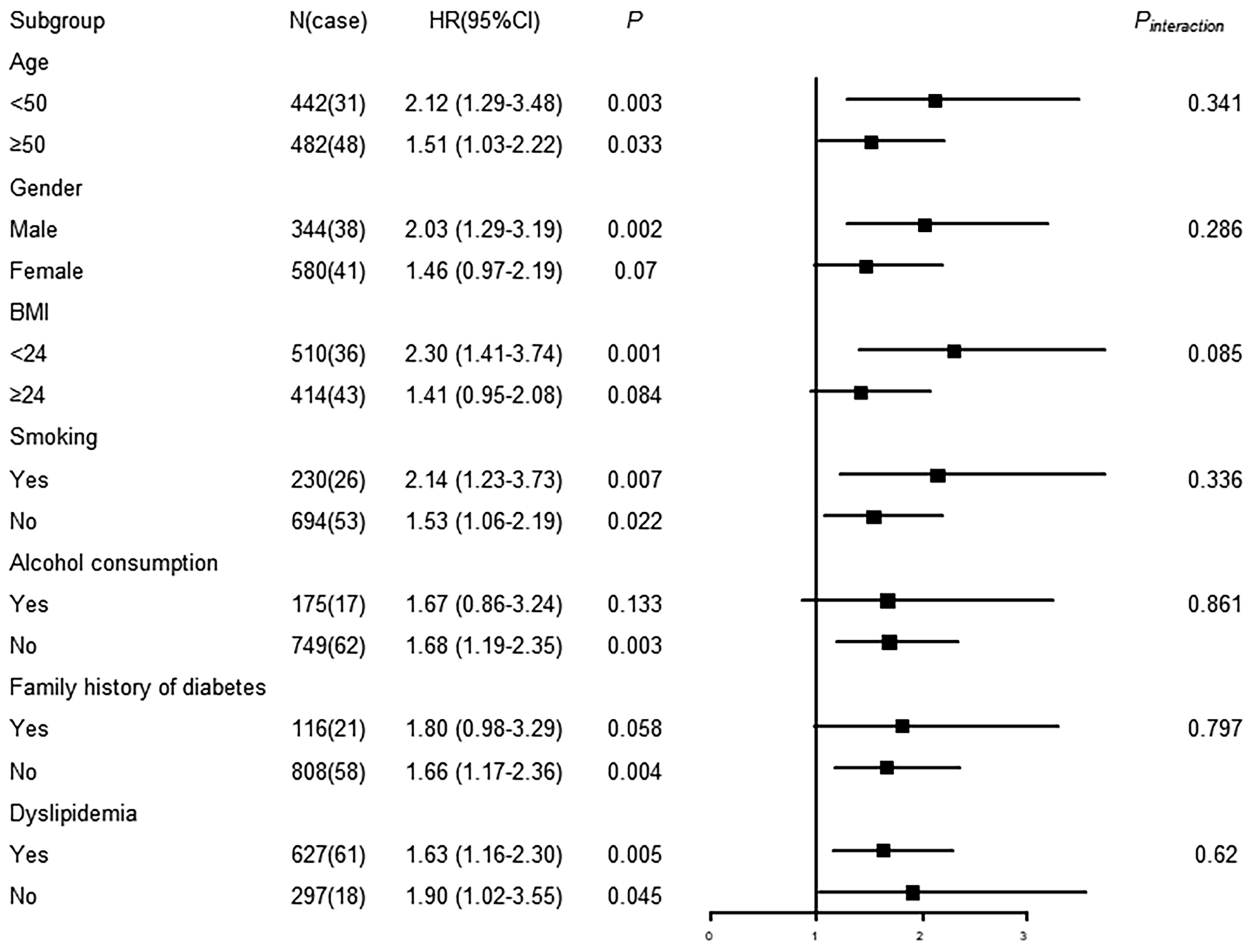
Subgroup analysis of associations between RvD2 and incident T2DM.

As the results shown in **Figure 4**, the association between RvD1 and T2DM was significant in the subgroups of ≥ 50 years, female, BMI <24 kg/m^2^, smokers, non-alcoholic consumers and dyslipidemia. Furthermore, a significant interaction was observed between RvD1 and BMI in relation to incident T2DM (*P*_interaction_ = 0.026). As shown in **Figure 5**, a significant association between RvD2 and T2DM risk was found in males (HR: 2.03, 95% CI: 1.29-3.19), participants with lower BMI (<24 kg/m^2^) (HR: 2.30, 95% CI: 1.41-3.74), non-alcoholic consumers (HR:1.68, 95%CI:1.19-2.35), and those without a family history of diabetes (HR:1.66, 95%CI: 1.17-2.36). However, none of the covariates modified in the association between RvD2 and the risk of T2DM.

**Table 4** showed the time-dependent ROC curve indicating the predictive values of RvD1 and RvD2 for the risk of T2DM based on the CDRS model. Although the predictive values of four models for T2DM risk decreased over time, the AUROCs of four models for predicting future T2DM risk fluctuated only within a narrow range over 3- to 7-year of follow-up period (CDRS: 0.836-0.824; CDRS+RvD1: 0.840-0.826; CDRS+RvD2: 0.839-0.827; CDRS+RvD1+RvD2: 0.842-0.828). Among them, the “CDRS+RvD1+RvD2” model exhibited the best predictive value for predicting the 3-year, 5-year and 7-year risk of T2DM probability.

**Table 4:** Best threshold and areas under the time-dependent receiver operating characteristic curves for different models predicting future T2DM risk.

	3-years	5-years	7-years
	AUC (best threshold)	AUC (best threshold)	AUC (best threshold)
CDRS	0.836 (0.532)	0.830 (0.524)	0.824 (0.514)
Model 1	0.840 (0.547)	0.833 (0.527)	0.826 (0.510)
Model 2	0.839 (0.548)	0.833 (0.518)	0.827 (0.505)
Model 3	0.842 (0.547)	0.835 (0.524)	0.828 (0.509)

CDRS includes age, BMI, waist circumference, dyslipidemia, hypertension, FPG and family history of diabetes.

Model 1 includes age, BMI, waist circumference, dyslipidemia, hypertension, FPG, family history of diabetes and RvD1.

Model 2 includes age, BMI, waist circumference, dyslipidemia, hypertension, FPG, family history of diabetes and RvD2.

Model 3 includes age, BMI, waist circumference, dyslipidemia, hypertension, FPG, family history of diabetes, RvD1 and RvD2.

AUC, area under the curve; CDRS, Chinese CDC T2DM prediction model; T2DM, type 2 diabetes mellitus.

## 4 Discussion

To the best of our knowledge, this study is the first work to assess the prospective association of D-series resolvins (RvD1 and RvD2) with subsequent 7-year risk of T2DM at the population level. The following key findings were identified: 1) higher concentrations of RvD1 and RvD2 are associated with a higher risk of developing T2DM; 2) BMI has a significant effect modification in the association between RvD1 and future T2DM probability; 3) the “CDRS+RvD1+RvD2” model has a better ability to predict the 3-year, 5-year and 7-year risk of T2DM. These findings fill a critical gap by providing evidence for the influences of RvD1 and RvD2 on the subsequent risk of T2DM at the population level.

While most cellular studies and animal models have reported protective effects of RvD1 and RvD2 on T2DM development and insulin resistance (23–25), several previous studies partially support our present findings (26–28). First, in non-obese diabetic mouse models, Parashar et al. observed that both plasma RvD1 and RvD2 were elevated in female mice after disease onset (27). Possible mechanisms for increased RvD1 and RvD2 in T2DM include increased expression of the enzymes 5-lipoxygenase, which is involved in the synthesis of the E- and D- series resolvins (28). Moreover, Barden et al. found that the levels of the SPMs of 18-HEPE, 17-HDHA, RvD1 and 17R-RvD1 were all significantly elevated in T2DM patients compared with healthy controls, even after adjustment for age and gender (26). Second, a number of studies have reported that the anti-inflammatory molecules may have different effects on the development of T2DM at different stages of the disease, which may partially explain our findings. For example, Moustafa et al. reported that IL-10 levels were higher in the prediabetes individuals than in healthy controls (29). However, Bashir et al. suggested that serum levels of IL-10 were lower in T2DM cases compared with healthy controls (30). Last, our present results showed that both plasma RvD1 and RvD2 concentrations were positively correlated with plasma hs-CRP levels at baseline. It is therefore reasonable to speculate that elevated RvD1 and RvD2 levels may be a homeostatic response to an ongoing inflammation, which should be a feedback regulation that would contribute to the balance between the onset and resolution of inflammation.

In this study, the results of subgroup analysis showed that RvD1 and RvD2 were significantly associated with incident T2DM in the participants with BMI < 24 kg/m^2^. Moreover, there was a significant effect modification of BMI on the association between RvD1 and future T2DM probability. In animal models, Echeverría et al. reported that high-fat diet-fed mice induced body weight gain, liver steatosis, TG accumulation, up-regulation of pro-inflammatory markers, and accompanied by increased hepatic levels of RvD1 and RvD2 and resolvins E1/2, suggesting resolvins synthesis was enhanced and constituted a possible adaptive mechanism to counteract inflammation expansion in obesity animals (31). Indeed, our present results also showed a positive correlation between plasma RvD1 and BMI, and between plasma RvD2 and BMI. Moreover, the HRs of the RvD1 and RvD2 associations with T2DM were lower in the overweight/obese participants than in those with BMI less than 24 kg/m^2^ (1.24 *vs* 3.79 for RvD1; 1.41 *vs* 2.30 for RvD2). On the other hand, Mas et al. have reported that inadequate SPM in chronically inflamed obese white adipose tissue (WAT) of patients might be due to a lower intake of DHA and EPA (32). In addition, the upregulation levels of SPM metabolizing enzymes such as 15-PGDH and eicosanoid oxidoreductases in obese WAT was also associated with the inadequate SPM levels (15, 33). Therefore, the findings of our study and of previous studies suggest that the effects of RvD1 and RvD2 on T2DM risk should be further investigated in overweight/obese populations.

Until now, the complicated interplay between RvD1 and RvD2 and traditional T2DM risk factors remains unclear. In this study, we observed that RvD1 and RvD2 were significantly associated with T2DM risk in females and males, respectively. Several previous studies have suggested that there may be gender differences in the production of RvD1 and RvD2 (27, 34–37). For example, results from animal models suggested that RvD1 levels were significantly reduced in aged male mice, but less so in aged female mice (34). In addition, Rathod et al. suggested that female sex protects against the endothelial dysfunction induced by a mild systemic inflammatory response and that this protection likely relates to an accelerated resolution of inflammation (35). However, other studies have shown that plasma RvD1 levels are relatively lower in females than in males, but there is no difference in plasma RvD2 between the sexes (36). For example, *Shum M et al.* reported that cystic fibrosis airway epithelium in the abnormal resolution of inflammation and with worse pulmonary outcomes in women (37). In addition to gender difference, we also found that RvD1 and RvD2 were significantly associated with incident T2DM in non-alcoholic consumers. Therefore, additional epidemiological studies are needed to investigate the complex synergistic effects between RvD1 and RvD2 and traditional T2DM risk factors.

Time-dependent ROC curves have been widely used to assess the predictive power of diagnostic markers for time-dependent disease outcomes (38). In the current study, we also evaluated the predictive value of RvD1 and RvD2 for predicting 3-year, 5-year and 7-year T2DM risk based on the traditional CDRS model. As expected, the model of “CDRS+RvD1+RvD2” exhibited a higher AUROC throughout the follow-up period, as compared to the other three models. Of noted, although the AUROCs of “CDRS+RvD1+RvD2” used for T2DM prediction fluctuated only within a narrow range, time-dependent ROC analysis showed that the predictive value of “CDRS+RvD1+RvD2” model for T2DM risk decreased over time. Given that the balance between the occurrence and resolution of inflammation is dynamic and a steady state is maintained through a complex continuum of feedback, future work should explore the trajectories of RvD1 and RvD2 during follow-up and their impact on the risk of T2DM development.

The strength of the prospective design is that it enables the collection of information on exposure variables prior to disease onset, which reduces the potential recall bias and the risk of reverse causation. Moreover, we investigated the predictive value of RvD1 and RvD2 for predicting 3-year, 5-year and 7-year T2DM risk using the time-dependent ROC. However, our study has several limitations that warrant discussion. First, considering that the different results observed in our population study and the previous study based on cell and animal models, the effects of RvD1 and RvD2 on the development of T2DM should be further elucidated in the future. Second, the plasma levels of RvD1 and RvD2 were measured only at the baseline. The levels may have changed over time before the incidence of the disease. Therefore, it should be examined in the future whether fluctuations in the RvD1 and RvD2 levels might be associated with T2DM risk. Third, it would be interesting to investigate the effect of diet (e.g., fish oil or omega-3 fatty acids) on the association of RvD1 and RvD2 with T2DM risk; however, we did not have any data on diet in the current work. Fourth, hs-CRP was selected as the inflammatory marker in this study. Given that hs-CRP is a downstream marker of inflammation, further studies are required involving a wider spectrum of inflammatory biomarkers such as fibrinogen, IL-6, and tumor necrosis factor-alpha. In addition, the levels of resolvins precursors and their effects on the development of T2DM need to be further analyzed. Fifth, the relatively small sample size of the subgroup, particularly the number of the participants with elevated hs-CRP levels, limited the ability of our analysis to identify traditional T2DM-related factors with small effect modification. Last, this was not a nationally representative sample, and all participants were over 35 years of age. Therefore, caution should be exercised when interpreting our findings in younger and other ethnic populations.

In summary, we examined the D-series resolvins (RvD1 and RvD2) and the risk of T2DM in a community-based prospective cohort study. We are the first to report that higher plasma RvD1 and RvD2 concentrations are associated with a higher risk of T2DM at the population level. The findings will broaden our understanding of the pathobiology and mechanisms of T2DM development. Future studies are warranted to investigate the effect of RvD1 and RvD2 fluctuations on T2DM risk during long-term follow-up.

## Data availability statement

The original contributions presented in the study are included in the article/**Supplementary Material**, further inquiries can be directed to the corresponding author/s.

## Ethics statement

The studies involving human participants were reviewed and approved by the ethics committee of Suzhou Industrial Park Centers for Disease Control and Prevention. The patients/participants provided their written informed consent to participate in this study.

## Author contributions

HZ and CD contributed to the conception and design of the study. QS, JW, YJ and CD contributed to manuscript drafting. QS, JW, YJ, SW and HZ contributed to the statistics analysis. QS, JW, YJ, JL, JJ, JZ, KL and XC contributed to the acquisition of data. QS, HZ and CD contributed to critical revisions of the manuscript. All authors contributed to the article and approved the submitted version.

## Supplementary material

Supplementary Figure 1 Kaplan-Meier analysis on the cumulative incidences for T2DM stratified by quartiles of the concentrations of RvD1 **(A)** and RvD2 **(B)**, respectively. Differences between cumulative incidences for RvD1 and RvD2 quartiles were tested using the log-rank test.

Supplementary Figure 2 Restricted cubic splines analysis on the linearity of RvD1 **(A)** or RvD2 **(B)** for the risk of T2DM. Analyses were multivariable-adjusted using 5 knots (located at the 5th, 25th, 50th, 75th, and 95th percentiles).
