# Supplementary figures and images for: Influences of resolvin D1 and D2 on the risk of type 2 diabetes mellitus: a Chinese community-based cohort study
Source: Front Immunol. 2023 Jun 2;14:1143456. doi: 10.3389/fimmu.2023.1143456 (PMC10272361; doi:10.3389/fimmu.2023.1143456)

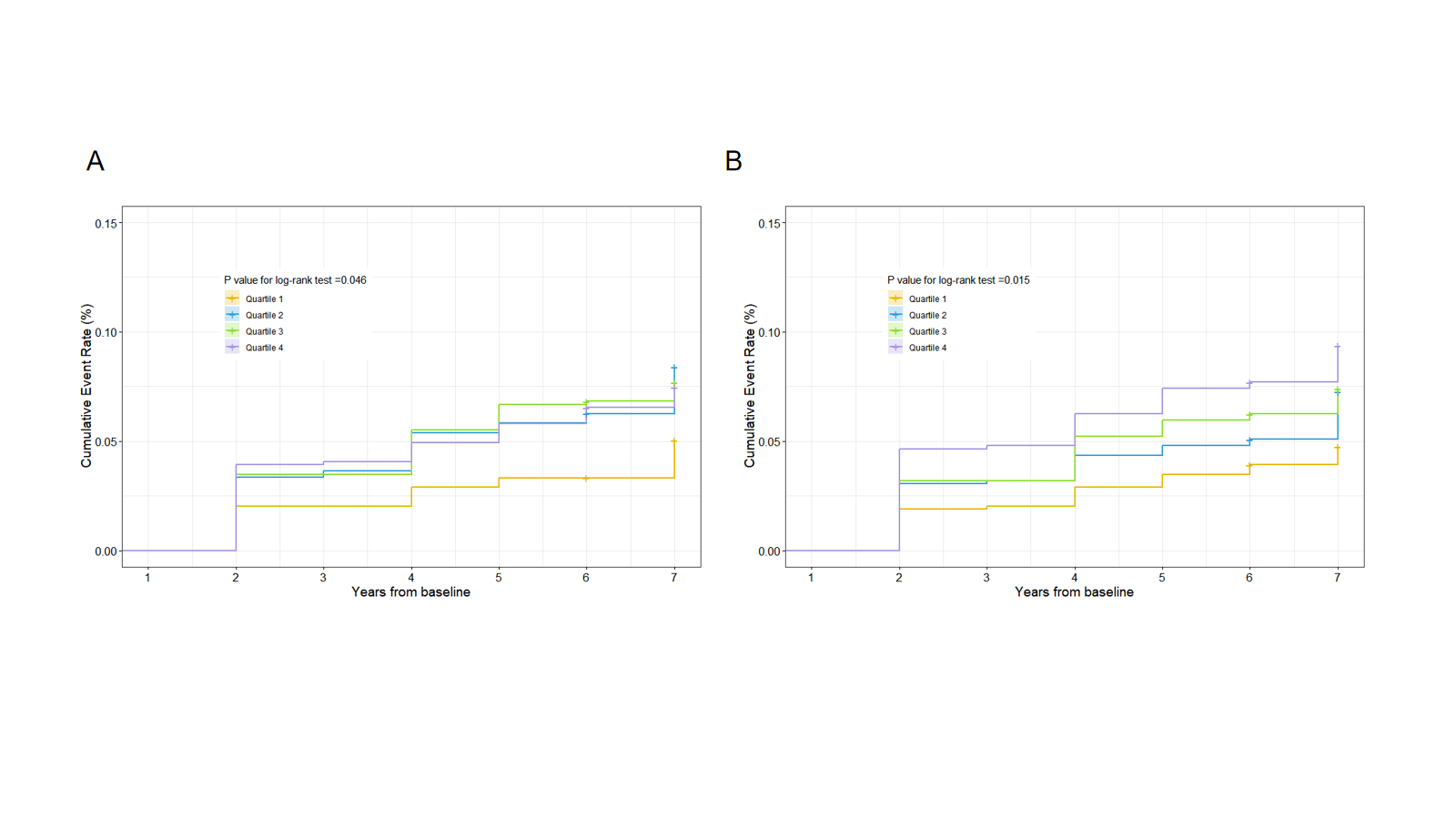

Supplement: Supplementary Figure 1 — Kaplan-Meier analysis on the cumulative incidences for T2DM stratified by quartiles of the concentrations of RvD1 (A) and RvD2 (B), respectively. Differences between cumulative incidences for RvD1 and RvD2 quartiles were tested using the log-rank test. [file Image_1.tif]

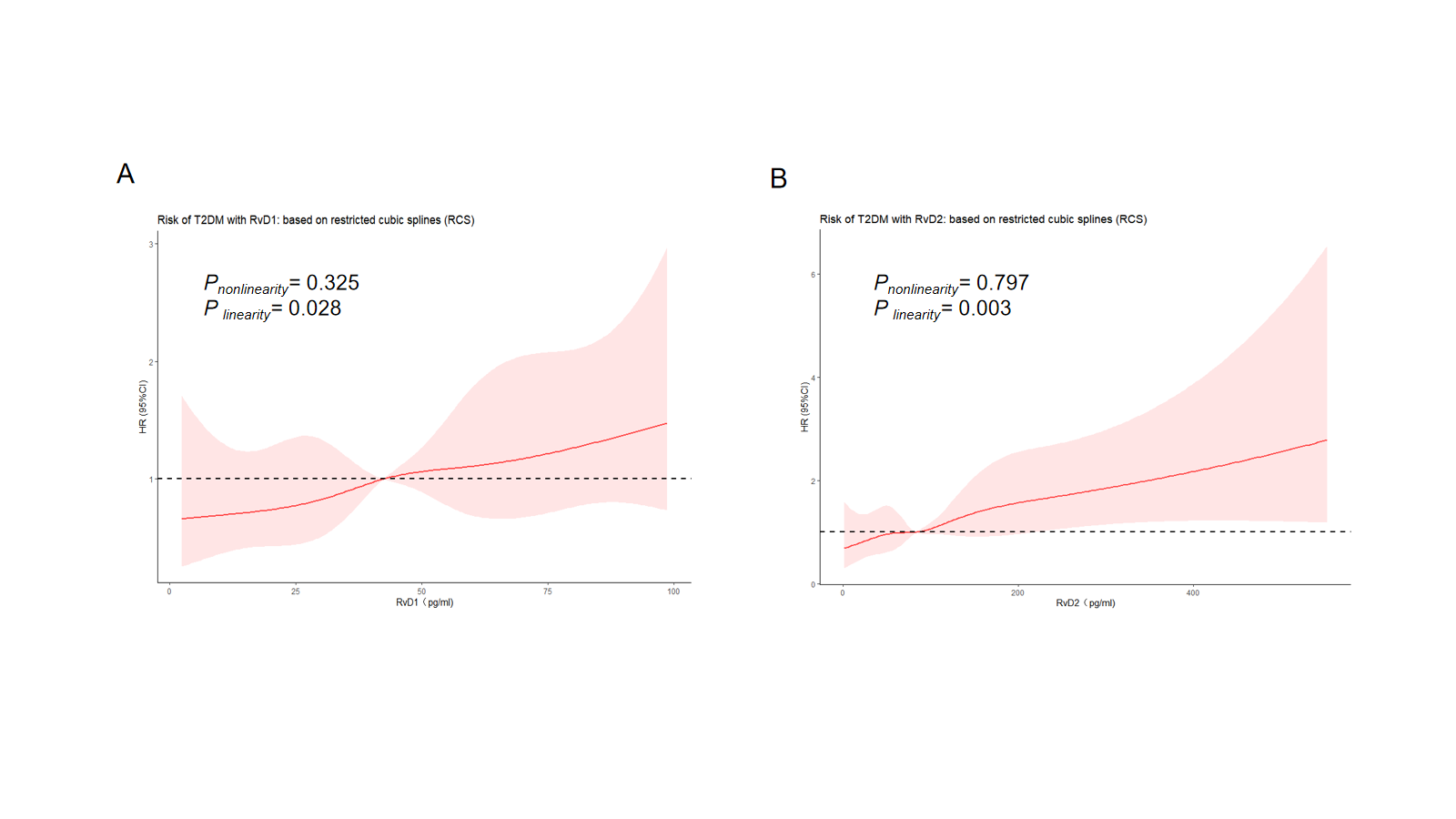

Supplement: Supplementary Figure 2 — Restricted cubic splines analysis on the linearity of RvD1 (A) or RvD2 (B) for the risk of T2DM. Analyses were multivariable-adjusted using 5 knots (located at the 5th, 25th, 50th, 75th, and 95th percentiles). [file Image_2.tif]
